# Potential role of mindfulness in the adjuvant treatment of atopic dermatitis

**DOI:** 10.1002/ski2.423

**Published:** 2024-07-23

**Authors:** Filomena Russo, Anna Dattolo, Donatella Sordi, Anna Rita Giampetruzzi, Ornella de Pità, Annarita Panebianco, Barbara Cocuroccia

**Affiliations:** ^1^ Istituto Dermopatico dell'Immacolata Istituto di Ricovero e Cura a Carattere Scientifico Rome Italy

## ETHICS STATEMENT

Not applicable.

## PATIENT CONSENT

Not applicable.

Dear Editor,

Practices, such as meditation, that 50 years ago seemed like new age inventions have, over time, had widespread influence on our lives, attracting the attention of the scientific community, which has attested to their validity beyond any scepticism. Today, meditation and other mind–body practices, such as yoga and mindfulness, are becoming increasingly popular.^1^ Mindfulness is a technique that focuses attention on the present moment and is based on the non‐judgemental acceptance of one's thoughts and emotions. Several studies have highlighted the possible role of meditation and coping mechanisms when dealing with flares in patients with atopic dermatitis (AD), by improving the management of stress during flare‐ups and the intensity of itching and the associated sleep disturbances.^2^

Although itching is the cardinal symptom of AD,^3^ only a few studies have focused on the brain mechanisms related to it, suggesting that the brain is a possible therapeutic target and whether psychological interventions could have an anti‐itch effect in AD.^2^ Therefore, understanding and treating the neurosensory aspects of itch may be important facets in the management of chronic itch.

Since 1994, several brain imaging studies, conducted using functional magnetic resonance (fMRI), have revealed the presence in affected patients of functional and structural anomalies in the brain areas associated with the processing of itch (which include the somatosensory cortex, cingulate cortex, medial parietal cortex, insular cortex and motor cortex).^4^ The cingulate cortex has been associated with cognition/evaluation of itchy stimuli and/or the need to scratch. The medial parietal cortex has been associated with subjective sensations of itching and pain and has also been associated with memory and attention faculties. Finally, the activation of the motor cortex during itchy stimuli events can additionally reflect on both the motor preparation for scratching and the need to scratch.

Notably, Ishiuji et al. reported a significant correlation between percentage changes in brain activation of the anterior cingulate cortex and contralateral insula and itch intensity, as well as with disease severity in AD patients.

In these areas the subjective sensation of itching is evaluated in its intensity and translated into the need to scratch by activating memory mechanisms, which can also make the observation of the scratching of others feel like an itchy stimulus.

Using MR, recent studies have shown that meditation can deactivate these areas of the brain and prevent the itchy sensation from becoming overwhelming while practicing mind training.^2^ Therefore, it might be reasonable to assume that psychological interventions aimed at reducing stress, such as mindfulness, would also have a positive effect on itching, altering the activity of the brain structures related to it.

To date, despite the importance the treatment of the neurosensory aspects of pruritus, this topic has not received as much attention as the dermatologic pathophysiology. The world of medicine has produced revolutionary healthcare benefits through advances in pharmacotherapies but now faces enormous challenges in battling stress‐related diseases. Going forward, it would be of great interest to investigate the effects of psychological interventions, such as mindfulness‐based stress reduction in patients with chronic pruritus, using robust and well‐controlled prospective studies.

## CONFLICT OF INTEREST STATEMENT

F. Russo acted as a speaker and consultant for Abbvie, Leopharma, Novartis and Sanofi, D. Sordi, A.R. Giampetruzzi, O. De Pità, B. Cocuroccia have no conflicts of interest.

## AUTHOR CONTRIBUTIONS

**Filomena Russo**: Conceptualization (equal); investigation (equal); methodology (equal); writing – original draft (equal); writing – review & editing (equal). **Anna Dattolo**: Writing – review & editing (equal). **Donatella Sordi**: Investigation (equal); writing – review & editing (equal). **Anna Rita Giampetruzzi**: Conceptualization (equal); writing – review & editing (equal). **Ornella de Pità**: Conceptualization (equal); writing – review & editing (equal). **Annarita Panebianco**: Validation (equal). **Barbara Cocuroccia**: Conceptualization (equal); writing – review & editing (equal).

## Data Availability

Data derived from public domain resources.
